# Ligand-Binding-Site Structure Shapes Allosteric Signal Transduction and the Evolution of Allostery in Protein Complexes

**DOI:** 10.1093/molbev/msz093

**Published:** 2019-04-19

**Authors:** György Abrusán, Joseph A Marsh

**Affiliations:** MRC Human Genetics Unit, Institute of Genetics and Molecular Medicine, University of Edinburgh, Edinburgh, United Kingdom

**Keywords:** quaternary structure, evolution of allostery, critical residues, communities, ligand binding

## Abstract

The structure of ligand-binding sites has been shown to profoundly influence the evolution of function in homomeric protein complexes. Complexes with multichain binding sites (MBSs) have more conserved quaternary structure, more similar binding sites and ligands between homologs, and evolve new functions slower than homomers with single-chain binding sites (SBSs). Here, using in silico analyses of protein dynamics, we investigate whether ligand-binding-site structure shapes allosteric signal transduction pathways, and whether the structural similarity of binding sites influences the evolution of allostery. Our analyses show that: 1) allostery is more frequent among MBS complexes than in SBS complexes, particularly in homomers; 2) in MBS homomers, semirigid communities and critical residues frequently connect interfaces and thus they are characterized by signal transduction pathways that cross protein–protein interfaces, whereas SBS homomers usually not; 3) ligand binding alters community structure differently in MBS and SBS homomers; and 4) except MBS homomers, allosteric proteins are more likely to have homologs with similar binding site than nonallosteric proteins, suggesting that binding site similarity is an important factor driving the evolution of allostery.

## Introduction

Proteins are dynamic entities, and usually experience conformational changes upon binding their ligands (Pabis et al. 2018). Allostery is a special case of conformational change induced by ligand binding, which is characterized by information transfer within proteins (see recent reviews, Dokholyan 2016; Guo and Zhou 2016a; Schueler-Furman and Wodak 2016; Wagner et al. 2016). Allosteric proteins can have two types of ligands: orthosteric ligands that are the substrate, and allosteric ligands that bind a different site, and regulate the activity of the orthosteric site. Allosteric ligands can be activators, inhibitors or regulators, depending on their effect on the orthosteric site, and are usually required for the normal functioning—or inhibition of function—of allosteric proteins. Allostery has large practical importance for drug design, because allosteric sites are druggable, and in the case of proteins with structurally similar orthosteric sites (e.g., in protein kinases, with similar ATP-binding sites [Kornev and Taylor 2015; Dokholyan 2016]), drugs that bind allosteric sites allow targeting-specific proteins, without the side effects of molecules that bind their unspecific orthosteric site.

Since its discovery five decades ago (Monod et al. 1963; Koshland et al. 1966), several mechanisms of allostery have been proposed, from the classic MWC (Monod et al. 1963), KNF (Koshland et al. 1966), and Cooper and Dryden (Cooper and Dryden 1984) models to the modern, ensemble-based view of allostery (Motlagh et al. 2014). Current views of allosteric signal transduction are based on dynamics (Kumar et al. 1999), and can be fundamentally grouped into two types: the “domino model” and the “violin model” (Kornev and Taylor 2015; Wagner et al. 2016). In the domino model, a distinct set of residues form a pathway within the protein between the allosteric and orthosteric site, and can transfer information, for example, by motions of the side chains of residues (Bedem et al. 2013). Although this mechanism of information transfer has been demonstrated in certain proteins (Lockless and Ranganathan 1999; Süel et al. 2003), it is likely to be much less common than the violin model, in which the motions of the entire protein are modulated by the binding of allosteric ligands at particular sites, similar to the modulation of sound of a violin on the fingerboard. (Note that several proteins are known to have more than one allosteric mechanism and pathway [Feher et al. 2014].) Here we focus on the violin model, using a relatively recent computational method of allosteric pathway identification: community analysis. Community analysis was first performed by Daily and Gray (2009) and Sethi et al. (2009). Using molecular dynamics (MD) and structure comparisons, these authors found that, in allosteric proteins, residues can be partitioned into “communities,” that is, groups of residues with correlated motions, that move as rigid bodies in the protein, and are connected by flexible “critical” residues, with high degrees of betweenness centrality. Subsequently, several studies have validated this approach experimentally (Rivalta et al. 2012; Farabella et al. 2014; McClendon et al. 2014; Aoto et al. 2016; Guo and Zhou 2016b; Zhong et al. 2017), showing that community analysis is an effective way of identifying allosteric signal transduction pathways (STPs) in proteins and protein complexes.

**Fig. 1.**
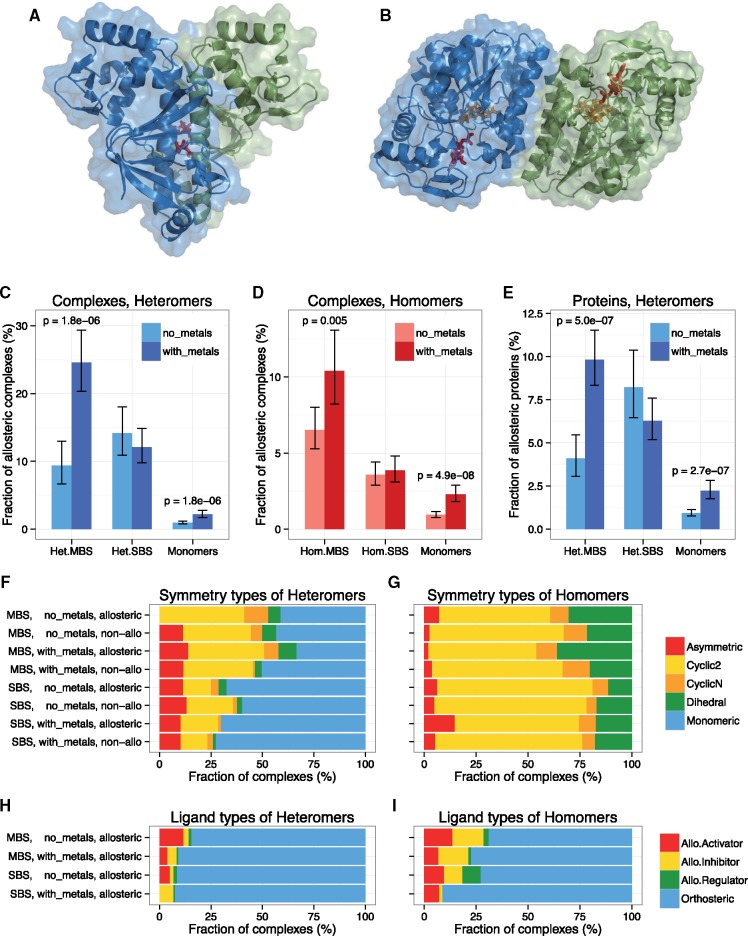
The frequency of allostery is influenced by the ligand type and the ligand-binding site. (*A*) Example of a homomer with MBS (global nitrogen regulator protein, NtcA, PDB ID: 3la3). (*B*) A homomer with SBS (UDP-galactose 4-epimerase, PDB ID: 5gy7). (*C*–*E*) In MBS complexes and monomers that bind metals, allostery is significantly more frequent than in such complexes without metals (see supplementary fig. S1, Supplementary Material online for the pattern with cofactors). Additionally, in MBS homomers and metal-binding MBS heteromers, allostery is significantly more frequent than in SBS complexes (see *P*-values in the text). In heteromers, the frequency of allosteric proteins is much lower than the frequency of allosteric complexes (*E*), and comparable to homomers, metal binding still has a significant effect in ones with MBSs. (*F* and *G*) In heteromers, the frequency of cyclic + dihedral symmetry is twice as high in MBS compared with SBS complexes (43.5% vs. 20.3%, *P* ≪ 0.005), whereas in allosteric MBS homomers, dihedral symmetry is more frequent than in nonallosteric ones (32.7% vs. 21.1%; *P* = 0.0046). Generally, however, there are no dramatic differences in the symmetry types of allosteric and nonallosteric complexes (note that “monomeric” symmetry indicates heteromers where every protein in the complex has only a single copy). (*H* and *I*) The vast majority of allosteric proteins are crystallized only with their orthosteric ligand, both in the case of homomers and heteromers. (All tests are tests of proportions, whiskers are 95% CIs.)

In this work, we use community analysis to test whether there are general relationships between the ligand-binding-site structure, the protein complex topology, and the structure of allosteric pathways. We have recently found that the structure of ligand-binding sites has profound consequences for the evolution of function and quaternary structure of protein complexes (Abrusán and Marsh 2018). We grouped ligand-binding sites into two categories: multichain binding sites (MBSs, fig. 1*A*), which contain residues from more than one protein chain in a complex, and single-chain binding sites (SBSs, fig. 1*B*), that are restricted to a single protein chain. Homomers with MBSs are characterized by much slower evolution of new functions, more similar binding sites and ligands. In the case of cofactor and metal-binding sites, they are also characterized by less variable quaternary structure than homomers with SBSs, or monomers (Abrusán and Marsh 2018), indicating different strength of selection in the two types of complexes.

Since the binding sites of homologous MBS homomers are much more similar to each other than the binding sites of SBS homomers, we hypothesized that the activity of MBS complexes must be regulated more tightly to avoid off-target activity. Thus, nature essentially faces a similar specificity problem with the activity of MBS complexes as the research and pharmaceutical community in the development of specific drugs for targets with unspecific binding sites. A solution for this problem might be regulation by allostery, which is supported by our observation that complexes with MBSs are significantly more flexible than complexes with SBSs (Abrusán and Marsh 2018), as flexibility is a key requirement of dynamics-driven allostery. It has also been pointed out (but not tested quantitatively) by Changeux (2012) that ligands binding several protein chains are common in allosteric protein complexes. In this article, we address the following questions: 1) Are MBS complexes more likely to be allosteric than SBS complexes? 2) Are there qualitative differences in the topology and functioning of allosteric pathways in MBS and SBS complexes? 3) Does the structural similarity of binding sites influence the frequency of allostery?

## Results

### The Frequency of Allostery Depends on Ligand Type and Binding Site Type

To test whether the frequency of allostery is different in different protein complexes, we analyzed proteins in the Allosteric Database (ASD, v.3) (Shen et al. 2016), a comprehensive, manually curated database of allosteric proteins and modulators, that currently contains data on 1,473 allosteric proteins. Protein complexes were divided into MBS and SBS complexes, using a comparable procedure as in Abrusán and Marsh (2018) (see Materials and Methods for details and an example of MBS homomers and SBS homomers on fig. 1*A* and *B*). Since we previously found that metal ions and cofactors are particularly important in the evolution and function of quaternary structure, we tested whether the presence of metal ions and cofactors influences the frequency of allostery. Our results show that in MBS complexes and monomers, allostery is significantly more common among the metal binding ones, whereas in SBS complexes, this is not the case (fig. 1*C*–*E*). In contrast, the frequency of allostery is considerably lower among cofactor binding heteromers than among the ones without cofactors, irrespective of the binding site type, although there is a trend but no significant difference in homomers (supplementary fig. S1*A*–*C*, Supplementary Material online). This suggests that some of the most studied examples of allostery like hemoglobin are actually atypical cases, in the sense that that cofactor binding proteins are generally less likely to be allosteric, probably due to their more rigid tertiary structure. In heteromers, although the frequency of allosteric complexes is higher than in homomers, the frequency of allosteric proteins is not (fig. 1*C* vs. *E*), indicating that in allosteric heteromers, frequently only some of their subunits are allosteric (although the incompleteness of ASD is likely to significantly contribute to this pattern). Additionally, in homomers, the frequency of allostery is significantly higher in MBS than in SBS complexes (fig. 1*D*, *P* < 0.005 for all four possible comparisons, tests of proportions; see also analyses below), whereas in the case of heteromers, only metal-binding MBS complexes have higher frequency of allostery than SBS complexes (fig. 1*C*, *P* < 0.005 for both possible comparisons, tests of proportions).

Since the symmetrical nature of protein complexes is thought to have a pivotal role in allostery (Changeux 2013), we tested whether there are consistent differences in the symmetry types of MBS and SBS complexes (fig. 1*F* and *G*, supplementary fig. S1*D* and *E*, Supplementary Material online). Our findings indicate that the differences in symmetry are not sufficient to explain the difference between MBS and SBS complexes. In heteromers, complexes with cyclic and dihedral symmetry are significantly (*P* = 1.25e-18, test of proportions) less frequent in SBS complexes (20.3%) than in MBS complexes (43.5%), and the frequency of “monomeric” symmetry (i.e., where all protein chains have a single copy in the complex) is higher; however, their higher frequency among MBS complexes without metals does not translate to a higher frequency of allostery (fig. 1*F*). In homomers, the frequency of dihedral symmetry is higher in allosteric than nonallosteric MBS complexes (32.7% vs. 21.1%; *P* = 0.0046, test of proportions), consistent with a previous report (Bergendahl and Marsh 2017), but generally we found no dramatic differences between the different complex types (fig. 1*G*). However, since the large majority of homomers are symmetric, and among the asymmetric ones quaternary structure assignment errors are much more frequent (Ahnert et al. 2015), the frequency of symmetry can be used only to a limited degree to test for any link between symmetry and allostery. Similarly, we found no dramatic differences in the frequency of allosteric modulators and orthosteric ligands (fig. 1*H* and *I*), indicating that systematic differences in the type of allostery, that is, activation vs. inhibition, or the presence of inactive or active (orthosteric ligand binding) forms in the PDB are unlikely to cause the observed higher frequency of allostery in MBS complexes.

### Allosteric MBS and SBS Complexes Have Different Patterns of Community Structure

When analyzing allostery in individual proteins, the computational method of choice is MD. However, MD simulations are time consuming, have size limitations, and are unsuitable for large-scale analyses. Since our analysis involves hundreds of structures, we used a much more computationally efficient method that is based on normal mode analysis (Sanejouand 2013). We characterized the allosteric pathways of protein complexes with STRESS (Clarke et al. 2016), a recently developed tool that uses elastic networks to detect correlated motions between residues, and to identify communities and critical residues in structures. Elastic network models are less accurate but orders of magnitude faster than MD simulations, and are generally able to qualitatively reproduce the community structure and dynamics of proteins obtained with MD simulations (Mishra and Jernigan 2018; Tekpinar and Yildirim 2018). Similar to most MD studies, STRESS identifies correlated motions of residues using their C-α atoms. It has been reported that using residue center of mass (c.o.m.) instead of C-α atoms is necessary to identify critical residues with experimentally verified allosteric function (VanWart et al. 2012) in MD simulations. Therefore, we modified STRESS, to use residue c.o.m., and performed all calculations using both the residue c.o.m. and C-α method (see Materials and Methods for details).

**Fig. 2.**
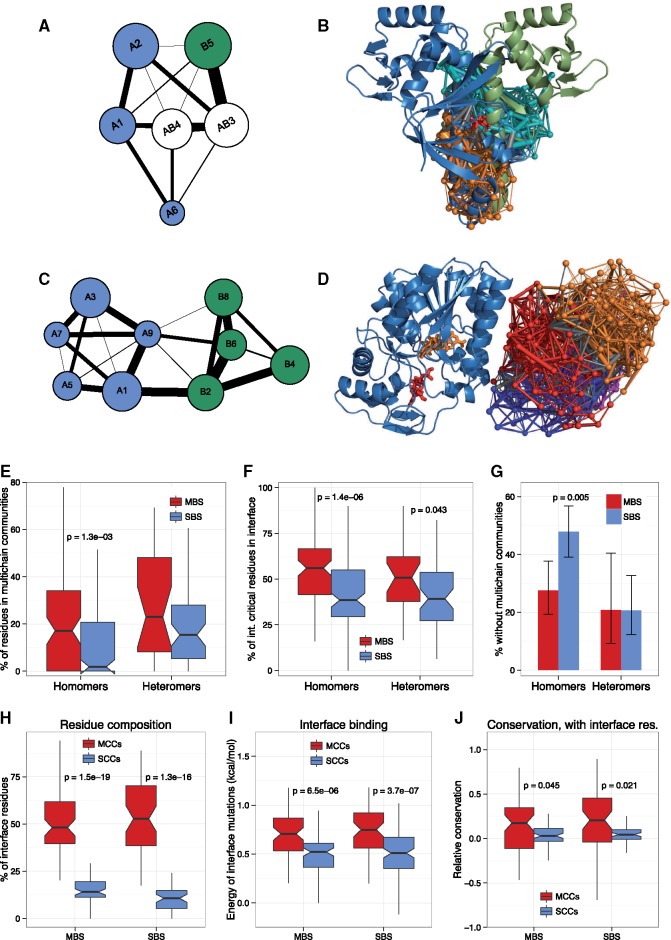
The allosteric pathways of MBS and SBS complexes are different. (*A*) Community structure of NtcA dimer. Two of its six communities, indicated with white, are MCCs, containing residues from both protein chains. (*B*) Localization of the NtcA MCCs in the homodimer (PDB ID: 3la3). (*C*) Community structure of UDP-galactose 4 epimerase. None of its nine communities are MCCs. (*D*) Visualization of the communities on chain B of the dimer (PDB ID: 5gy7). (*E*) In homomers, the percent of residues in MCCs is significantly higher than in heteromers. (*F*) The fraction of critical residues in protein–protein interfaces is significantly higher in MBS complexes than in SBS complexes, both in the case of homomers and heteromers. (*G*) In homomers, the fraction of complexes without MCCs is significantly lower in ones with MBSs. (*H*) In both MBS and SBS homomers, the fraction of interface residues in MCCs is significantly higher than in SCCs. (*I*) In MCCs, mutating individual interface residues to alanine results in significantly larger changes in the binding energy of interfaces than in SCCs, irrespectively of the type of the homomer. Note that only residues that are part of an interface were used, both in MCCs and SCCs. This indicates that in MCCs, the interactions of interface residues are much stronger than between interface residues of SCCs. (*J*) Conservation of residues in MCCs is significantly higher than in SCCs, although this is mostly due to their higher fraction of interface residues. (Tests of proportions on panel *G*, Wilcoxon tests on all other panels. Note that on all panels, *P* values are provided only for significant differences.)

We determined communities and interior critical residues for all protein complexes of the PDB where at least one of their subunits is present in the Allosteric Database and that have a biologically relevant ligand: 158 MBS homomers, 135 SBS homomers, 44 MBS heteromers, and 66 SBS heteromers. For every protein complex, a representative structure was chosen, which usually was its largest structure in the PDB (see Materials and Methods for the structure selection pipeline, and supplementary table S1, Supplementary Material online for the list of structures). For each structure, we determined its critical residues and communities with STRESS (see supplementary fig. S2, Supplementary Material online for a summary of the main parameters of the allosteric networks, and supplementary fig. S3, Supplementary Material online for examples of communities). STRESS models the protein complex as a network of residues, where each node is a residue, while edges are contacts between the residues. Communities are “modules” of the network that show much higher degree of correlated motion with residues of the same community than with residues of other communities, while critical residues are residues that connect communities, and have particularly high betweenness centrality in the network, and are particularly important in transmitting motions between communities. The comparison of communities of MBS and SBS complexes indicates that there are clear structural differences in the community structure of MBS and SBS homomers (fig. 2): most MBS homomers contain multichain communities (MCCs) that contain residues from multiple protein chains (see fig. 2*A* and *B* for an example), whereas SBS homomers mainly contain single-chain communities (SCCs, see fig. 2*C* and *D* for an example). The fraction of residues in MCCs is significantly different in MBS and SBS homomers (fig. 2*E*), as is the fraction of critical residues in protein–protein interfaces (fig. 2*F*). The frequency of complexes without MCCs is highest in SBS homomers, with no significant difference between MBS and SBS heteromers (fig. 2*G*).

These findings indicate a fundamental difference in the allosteric pathways of MBS and SBS homomers: in MBS homomers, there is much stronger communication between the different subunits of the complex than in SBS homomers, either through MCCs, or the higher frequency of critical residues in the interface. Additionally, the fraction of residues in MCCs of SBS homomers is probably overestimated, because in the case of complexes that have both an MBS and SBS structure in the PDB, the fraction of residues in MCCs is similar (supplementary fig. S4, Supplementary Material online); thus, some SBS complexes are likely to have an MBS form that is missing from the PDB.

The affinity for interaction between proteins in protein complexes form a continuum, from permanent complexes, that are stable during the lifetime of the proteins that form them, to transient complexes, which exist in an equilibrium between a complex and its dissociated subunits (Nooren and Thornton 2003a, 2003b). Permanent complexes are usually obligatory, that is, complex formation is necessary for their biological function, whereas transient complexes include various, typically heteromeric, and frequently nonobligatory interactions, like antibody–antigen, receptor–ligand, and enzyme–inhibitor interactions (Nooren and Thornton 2003). However, recent work indicates that in many, if not most homomeric complexes where quaternary structure was assumed to be functional, it may actually evolve neutrally, and not be related to function (Lynch 2013; Abrusán and Marsh 2018; Hagner et al. 2018); thus it is possible that such complexes are also frequently nonobligatory/transient. In a recent work that used primarily heteromers, it has been shown that the dynamics of a protein, and whether it has dynamic domains that cross interfaces, is a good predictor of the obligatory or nonobligatory nature of a protein complex (Soner et al. 2015). This suggests that among SBS complexes, which are characterized by fewer and smaller MCCs, the frequency of nonobligatory complexes is likely to be higher. Since nonobligatory complexes have generally less conserved interfaces (Mintseris and Weng 2005), we tested whether the conservation of interface residues is different between MBS and SBS complexes. We found no clear differences between the two complex types (supplementary fig. S5*A* and *C*, Supplementary Material online), and when relative interface area (interface area/surface area) is added as a covariate, even the nonsignificant trends disappear (supplementary fig. S5*B*, Supplementary Material online).

### Functional Characteristics of Multi- and Single-Chain Communities in Homomers

We tested whether MCCs are more likely to contribute to the information transfer across protein–protein interfaces of homomers than SCCs with two methods: by estimating the effect of mutations on interface binding, and conservation. MCCs and SCCs that do have interface residues contain different amounts of them, because MCCs contain residues from both chains of an interface (fig. 2*H*). To test whether protein–protein interfaces in MCCs are characterized by stronger binding energies, we mutated in silico each interface residue in a community to alanine (except alanines), and determined the effect of every mutation on the binding energy of the interface with FoldX (see supplementary fig. S6, Supplementary Material online and Materials and Methods). We found a highly significant difference between interface residues of MCCs and SCCs, irrespectively whether they originate from an MBS or SBS homomer: mutations in MCCs weaken the interface binding energy significantly more than mutations in SCCs (i.e., their effect on binding energy is more positive, fig. 2*I* and supplementary fig. S6, Supplementary Material online). Since interface residues in MCCs and SCCs might be characterized with different solvent accessibilities if they occupy different regions of the protein–protein interface, we also tested whether this pattern remains if we use the average solvent accessibility as covariate in the statistical analysis. Our results show that the difference between MCCs and SCCs remain highly significant in both in MBS and SBS homomers (supplementary fig. S7, Supplementary Material online), even if solvent accessibility is taken into account. This indicates that the stronger binding between interface residues in MCCs is not simply a by-product of their more buried location in the interfaces.

We also calculated the conservation levels of residues in MCCs and SCCs, which indicates that residues in MCCs are significantly more conserved than residues in SCCs (fig. 2*J*). However, the difference is primarily due to the higher conservation of interface residues; there is only a trend but no significant difference between the two when they are excluded (not shown). These findings indicate that the binding between interface residues of MCCs is stronger than of SCCs (which, by necessity represent binding between residues of more than one community), and therefore they are likely to contribute more to the information transfer between two protein chains than SCCs. Surprisingly, however, there is no qualitative difference in these patterns between MBS and SBS homomers that do have MCCs (fig. 2*E*–*G*).

### The Community Structure of Allosteric Complexes Scales with the Ratio of Interface and Surface Area

**Fig. 3.**
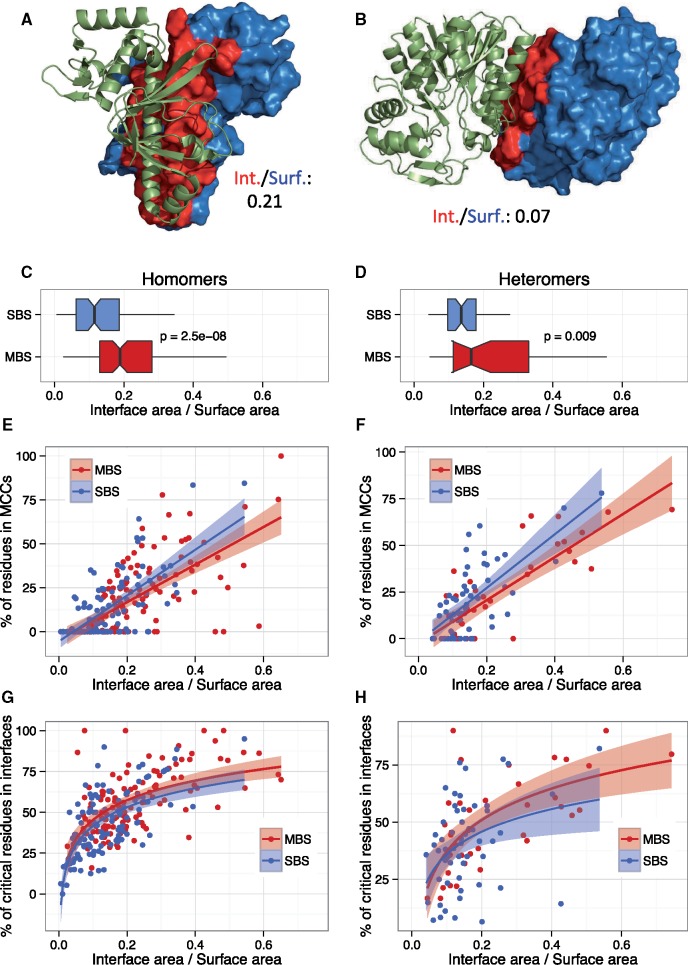
Community structure scales with the relative interface size of complexes. (*A* and *B*) Interface to surface ratios of NtcA and UDP-galactose 4 epimerase. Interface is highlighted with red, whereas the surface (shown only on one chain) with blue. (*C* and *D*) MBS complexes have significantly larger interface-to-surface ratios than SBS complexes, both in homomers and heteromers (Wilcoxon tests). (*E*–*H*) When interface-to-surface ratio is used as covariate, the percent of residues in MCCs (*E* and *F*), and the percent of critical residues in interfaces (*G* and *H*) scale similarly in allosteric MBS and SBS complexes (lines represent linear and logarithmic fit, with SE).

The lack of difference between the characteristics of MBS and SBS MCCs is likely to be at least partly caused by incorrectly classified SBS complexes (having no MBS form in the PDB, see supplementary fig. S4, Supplementary Material online). An alternative hypothesis is that the higher fraction of residues in MCCs and critical residues in interfaces (fig. 2*E*–*G*) might not be caused by a fundamental qualitative difference between the two complex types (i.e., MBS vs. SBS), but might be the result of simple topological differences between SBS and MBS complexes, for example, if different interface sizes result in stronger binding between the chains of MBS complexes. To test for this, we determined the interface and surface areas of all allosteric complexes (see fig. 3*A* and *B* for an illustration). We found that MBS complexes have significantly higher interface-to-surface ratios, both in homomers and heteromers (fig. 3*C* and *D*). Additionally, when the interface/surface ratio is used as a covariate, the fraction of residues in MCCs, and fraction of critical residues in interfaces scales similarly (fig. 3*E*–*H*), suggesting that this simple ratio—the relative interface area—might be sufficient to explain the observed differences.

### The Frequency of Allostery Is Higher in MBS Complexes with the Same Relative Interface Area

**Fig. 4.**
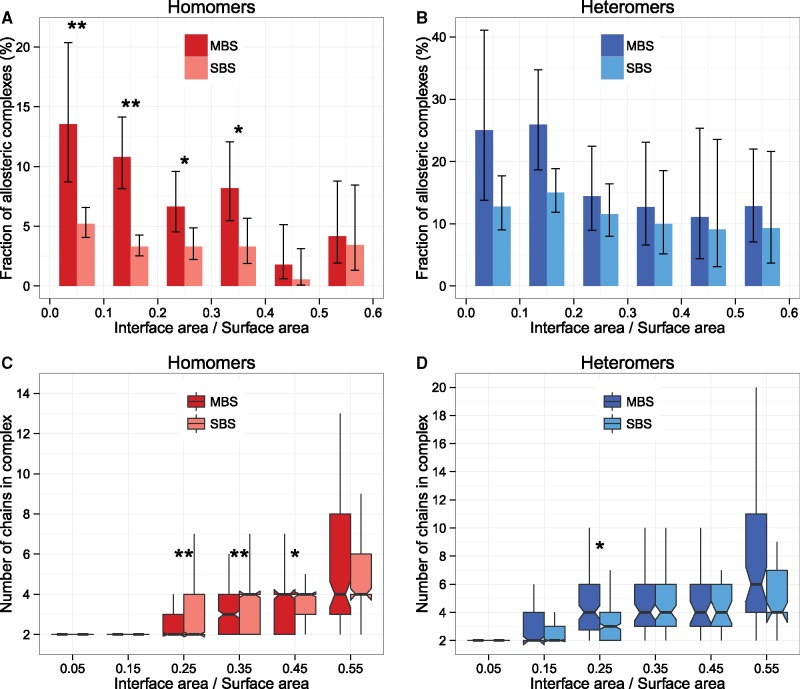
Allostery is more frequent in MBS homomers than in SBS homomers, irrespectively of their relative interface size. (*A* and *B*) The change of frequency of allostery with the interface-to-surface ratio (**P < 0.005, *P < 0.05, test of proportions, whiskers are 95% CI). (*C* and *D*) With increasing interface-to-surface ratio, the number of chains in protein complexes and also the size of complexes (supplementary fig. S4, Supplementary Material online) increases, both in homomers and heteromers (**P < 0.005, *P < 0.05, Wilcoxon tests). The declining trend seen on panels *A* and *B* is likely to be significantly influenced by experimental biases (and in consequence biases in the PDB): experimental methods like NMR have size limitations, MD simulations of large complexes are extremely time intensive, or dynamical properties are in general easier to determine for small complexes.

One prediction of the hypothesis that relative interface area is the key factor determining the differences in allostery between MBS and SBS complexes is that the frequency of allosteric proteins will be similar when scaled with this parameter. To test this, we determined the interface/surface ratio for every protein complex in the PDB, and tested whether the differences in the frequency of allostery between MBS and SBS complexes (see fig. 1) are simply the result of a higher ratio in MBS complexes. We found that this is not the case: in homomers, except for complexes with the highest interface/surface ratios, the fraction of allosteric complexes is significantly (2–3-fold) higher in MBS compared with SBS complexes (fig. 4*A*), but not in heteromers (fig. 4*B*). We also observe a clear reduction in the frequency of allostery with relative interface area (thus, the higher relative interface area of MBS complexes actually reduces the magnitude of the effect seen on fig. 1). However, this trend is likely to be the consequence of the limitations of the methods used in the investigation of allostery, rather than a real biological effect: for example, NMR spectroscopy and MD simulations of large complexes both have size limitations (Frueh et al. 2013). To test this, we examined how complex size and topological complexity (number of protein chains) scales with relative interface area. We found that both properties increase with relative interface area (fig. 4*C* and *D*, supplementary fig. S8*A* and *B*, Supplementary Material online). Thus, the difference in relative surface area between SBS and MBS homomers is not sufficient to explain the difference in the frequency of allostery (e.g., below relative interface area 0.2, there is no difference in complex size and chain number, despite the very large difference in the frequency of allostery, fig. 4*A*). Similarly, subunit flexibility increases with relative interface area, but we found no dramatic differences between MBS and SBS homomers with the same relative interface area (supplementary fig. S8*C* and *D*, Supplementary Material online), although MBS homomers have a tendency to be more flexible. Overall these results contradict the hypothesis that the larger interfaces of MBS homomers cause their higher frequency of allostery.

### Communities of MBS and SBS Homomers Respond Differently to Ligand Binding

**Fig. 5.**
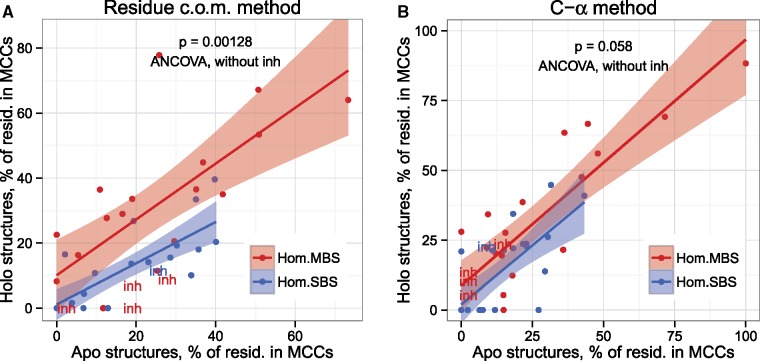
MBS and SBS homomers show different responses upon ligand binding. In SBS homomers, the number of residues in MCCs is reduced in the holo (ligand binding) structures, whereas in MBS homomers, their number is unchanged or slightly increased (ANCOVA, excluding structures with inhibitors. *P* indicates the significance of the categorical—binding site type—variable). Structures with inhibitors are indicated with “inh.” The residue c.o.m. method (*A*) indicates a significant difference, but not the C-α method (*B*). Additionally, in the case of residue c.o.m. method, structures crystallized with inhibitors (thus the ligand-binding form is their inactive form) are distinct from structures crystallized with their orthosteric ligands or activators, supporting the conclusions of VanWart et al. (2012), that the residue c.o.m. method is better in identifying biologically relevant residues in allosteric proteins.

Next, we tested whether the difference between MBS and SBS homomers might be caused by differences in their dynamical properties, that is, whether their community structures change similarly upon ligand binding. We identified 45 pairs of ligand-unbound (apo) and ligand-bound (holo) structures (22 MBS and 23 SBS) that satisfy the following criteria: are associated with the same study (PubMed ID), both have resolution better than 2.8 Å, and have similar size and number of subunits (see Materials and Methods for more details and supplementary table S2, Supplementary Material online, for the list of structure and their ligands). In the few cases where several holo structures were present with functionally different ligands (i.e., orthosteric and allosteric), more than one holo structure was used (see Materials and Methods). Using a similar procedure as described earlier, we identified the communities and critical residues of the pairs, and tested whether there is a consistent difference between the apo-holo structures of MBS and SBS complexes. Unlike in the previous analyses (figs. 2 and 3) where there was essentially no difference between the two methods, the residue c.o.m. and C-α methods perform differently (see fig. 5*A* vs. *B*), with the residue c.o.m. method showing a highly significant difference (*P* = 0.0012), whereas the C-α method is only at the edge of significance (*P* = 0.058) (fig. 5). We found that, using the residue c.o.m. method, the fraction of residues in MCCs in MBS homomers is higher in the holo structures than in SBS homomers (fig. 5) when the apo structure is used as a covariate. The fact that for the residue c.o.m. method, the few structures crystalized with their inhibitors (thus their holo form is the inactive form) have small numbers of residues in MCCs supports the conclusion of VanWart et al. (2012) that the residue c.o.m. method performs better in the identification of functional residues in allosteric structures (fig. 5*A*). These findings indicate that, despite the fact that the size of MCCs scales similarly with complex topology (fig. 3), the dynamical properties of MBS and SBS complexes are different.

### Human Complexes Having Homologs with Similar Binding Sites Are More Likely to Be Allosteric

**Fig. 6.**
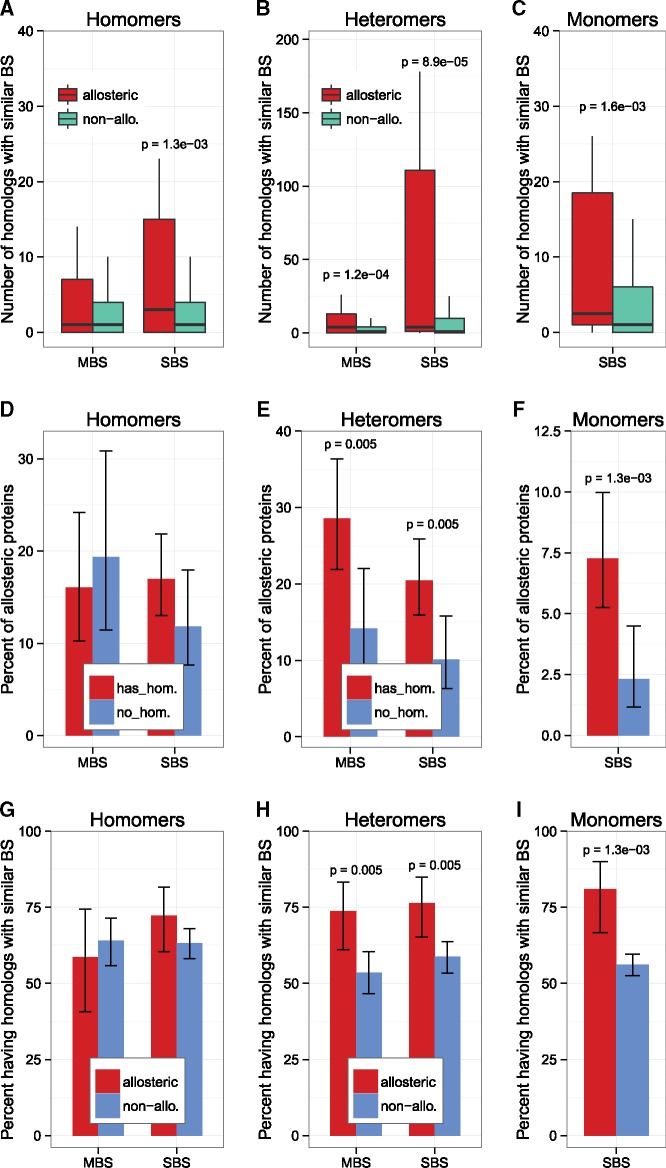
The presence of homologs with similar binding sites is a driving force of the evolution of allostery. (*A*–*C*) Except MBS homomers, proteins of allosteric complexes have many more homologs with similar BSs than nonallosteric proteins of the same complex type (*P* ≪ 0.05, one-sided Wilcoxon tests). (*D*–*F*) Except homomers, the frequency of allosteric proteins is ∼2× higher in proteins that have a homolog with a similar binding site, irrespectively of quaternary structure (one-sided tests of proportions; whiskers are 95% CI). In MBS homomers, proteins without homologs have a high frequency of allostery. (*G*–*I*) The opposite pattern, i.e. the frequency of homologs with similar binding sites in allosteric proteins shows a much smaller effect size (although similar significance): the percent of proteins having a homolog with a similar binding site is only 20–30% higher among allosteric proteins, irrespectively of their quaternary structure (one-sided tests of proportions, whiskers are 95% CI). These results indicate that primarily the presence of homologs drives the evolution of allostery, and not that allostery drives the emergence of homologs, although both processes are likely to contribute to the pattern.

Next, we tested whether the higher structural similarity of binding sites of MBS homomers (Abrusán and Marsh 2018) is the evolutionary driving force behind these differences, and the evolution of allostery (see Introduction). This hypothesis predicts that proteins/complexes that have homologs with similar binding sites are more likely to be allosteric than complexes that have no such homologs. Using the proteins of the human genome that have structural entries in the PDB, we identified the number of homologs for each protein in the human proteome, and also whether they have a similar binding site, using ProBiS (see Materials and Methods). We found that, with the exception of MBS homomers, allosteric proteins have significantly more homologs with similar binding sites than nonallosteric proteins of the same quaternary structure type (fig. 6*A*–*C*). These findings suggest that binding site similarity plays an important role in the evolution of allostery for the vast majority of proteins. An alternative hypothesis is that allostery contributes to the evolution of paralogs in the genome, as the functions of allosteric paralogs are less likely to overlap (due to regulation by allostery), or may diverge very rapidly. We tested which of these two hypotheses explain better the observations by examining whether: 1) proteins with homologs (having similar binding sites) are more likely to be allosteric than ones having no homologs, or 2) allosteric proteins are more likely to have homologs than nonallosteric ones.

We find that among proteins/complexes (except for MBS homomers) having at least one homolog with similar binding sites, the frequency of allostery is significantly, ∼100% higher than in proteins that have no homologs, irrespectively of quaternary structure (fig. 6*D*–*F*). This suggests that having homologs with similar binding sites play an important role in the evolution of allostery. The opposite pattern is much less pronounced: allosteric proteins have only 20–30% higher likelihood of having a homolog with a similar binding site compared to nonallosteric proteins (fig. 6*G*–*I*) in all quaternary structure types, except in MBS homomers. Thus the effect size is significantly weaker than of the previous hypothesis. (Note that due to the nature of the statistical test, the significances are identical.) These findings suggest that the structural similarity of ligand-binding sites is an important driving force behind the evolution of allostery, and that it is likely to shape allostery in most, if not all quaternary structure types, including monomers. Surprisingly, the only exceptions are MBS homomers, although this may be caused by the relatively low numbers of such proteins, and due to factors specific to the human genome. The alternative hypothesis, that allostery contributes to the emergence of paralogs in a genome, is also supported by the data (and both processes are likely to influence the frequency of allostery). However, the much larger effect size of homology on frequency of allostery (fig. 6*D*–*F* vs. *G*–*I*) suggests that primarily binding site similarity drives allostery, and not vice versa. In addition, the differences are not caused by consistent biases in metal or cofactor binding, because excluding metal and cofactor ligands does not change the pattern qualitatively (supplementary fig. S9, Supplementary Material online).

### Comparison of CA-COM Methods

**Fig. 7.**
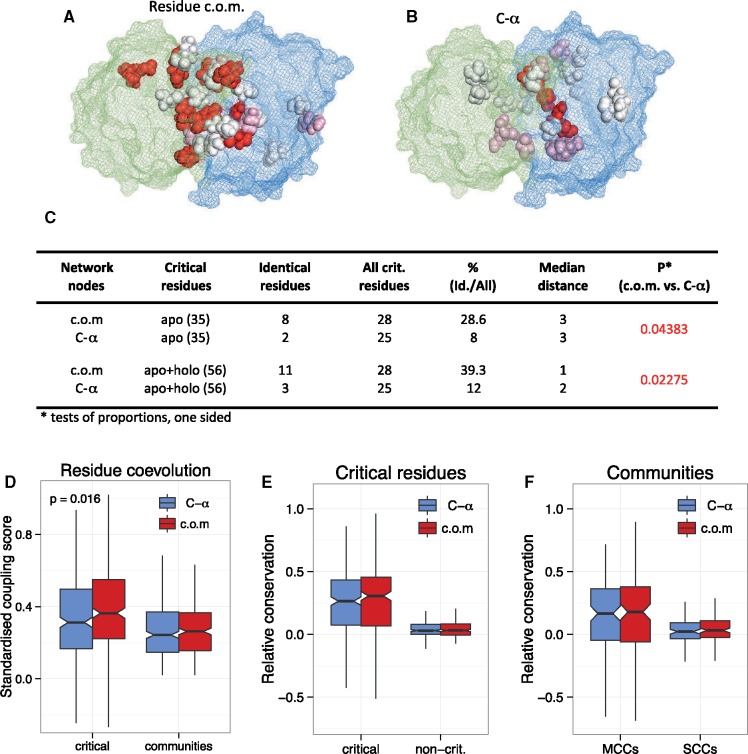
Comparison of the residue c.o.m. and C-α methods. (*A*) Critical residues of the HisH/HisF complex (imidazole glycerol phosphate synthase, PDB ID: 1gpw), identified with STRESS, using the residue c.o.m. method. Interior critical residues identified by STRESS are indicated by spheres. Red indicates residues that are identical to known critical residues identified by MD simulations of VanWart et al. (2012). Pink indicates residues located directly next to a known critical residue, and white indicates residues that are not in the direct proximity of a known critical residue. (*B*) Critical residues, identified by the default C-α method of STRESS, with similar color coding. (*C*) Statistical summary of the accuracy of critical residue identification in the HisH/HisF complex. The residue c.o.m. method performs better than the C-α method: a significantly higher fraction of STRESS critical residues are identical to the ones identified with MD (39% vs. 12%) by VanWart et al. (2012), both when only the critical residues of the apo structure (35 residues) or the combined set of apo + holo residues (56 residues) were used (one-sided tests of proportions). (*D*) The analysis of evolutionary couplings between critical and community residues indicate that critical residues are much more likely to coevolve than community residues, and that the residue c.o.m. method detects stronger couplings than the C-α method (*P* = 0.016, Wilcoxon test). (*E* and *F*) Despite the differences seen in HisH/HisF, the conservation of the critical residues and communities identified by the residue c.o.m. method is not higher than with the C-α method (Wilcoxon tests).

Finally, we performed a comparison of the residue c.o.m. and C-α methods for community and critical residue identification. First, using the apo structure of the imidazole glycerol phosphate synthase complex (HisH/HisF, PDB ID: 1gpw), we tested whether the critical residues identified by STRESS significantly overlap with the critical residues identified by VanWart et al. (2012). We found that the critical residues identified with the residue c.o.m. method (fig. 7*A*) have significantly higher overlap than the critical residues identified with the C-α method (fig. 7*B* and *C*). Approximately 40% of critical residues are identical to the residues identified by VanWart et al. (2012) when the residue c.o.m. method is used, compared with only 12% when the C-α method is used.

Next, using all homomeric and heteromeric complexes from our previous analyses, we tested whether there are differences in the coevolution of critical and community residues, using GREMLIN (Kamisetty et al. 2013). Coevolution between residues has been used to detect allostery for many years (Süel et al. 2003), although recent findings question whether information on allostery can be detected in such data (Anishchenko et al. 2017), and suggest that only residues that are relevant in folding and protein–protein interactions can be reliably detected with these methods. Our results show that critical residues are much more likely to coevolve than residues in communities (fig. 7*D*, residue c.o.m. method: *P* = 4.56e-11, Wilcoxon test; C-α method: *P* = 1.75e-05, Wilcoxon test). Moreover, the residue c.o.m. method detects a significantly, although not dramatically higher (*P* = 0.016) degree of coevolution between critical residues than the C-α method, suggesting that the former performs better in identifying them. Although resolving the debate whether coevolution between residues is suitable for identification of allosteric residues is beyond the scope of this work, we note that the high fraction of critical residues in protein–protein interfaces (figs. 2 and 3) means that our findings are consistent with both views. One likely cause of the discrepancy between the two views is that function evolves much faster than structure. This means that alignments that are sufficiently large to detect coevolution reliably must contain proteins with diverse functions, which results in weak correlations between residues that are functional but have no structural role. Alternatively, one could argue that alignments that can be used to reliably reconstruct the structure of the protein must contain proteins with diverse functions, in order to remove “noise” caused by functional, but structurally not relevant correlations. Finally, we tested whether the conservation of critical residues and communities identified by the two methods is different: aside from the highly significant difference between critical and noncritical residues (see also fig. 2), we found no significant differences between the two methods (fig. 7*E*–*F*).

Overall, our results support the conclusions of VanWart et al. (2012) that the use of residue c.o.m. should be preferred over the C-α method, for a number of reasons. In the case of HisH/HisF complex, it identifies significantly more residues that are identical to critical residues identified by MD. In the case of the analysis of apo-holo changes (fig. 5), the location of the structures with inhibitors suggests that it can identify biologically relevant patterns that the C-α method cannot. Similarly, the difference in evolutionary couplings also indicates that the residue c.o.m. method is more accurate, although we did not find a clear difference in the conservation scores of the two methods (fig. 7).

## Discussion

Our results show that ligand-binding-site structure is of major importance for allosteric signal transduction. First, MBS homomers and metal-binding MBS heteromers are much more likely to be allosteric than SBS complexes (figs. 1 and 4). Surprisingly, this does not seem to be associated with large differences in symmetry, and in the case of cofactor-binding heteromers, the frequency of allostery is significantly lower (supplementary fig. S1, Supplementary Material online). The lower frequency of allostery among cofactor-binding complexes is probably caused by their rigidity: cofactors stabilize the tertiary structure of proteins, and thus such proteins are more likely to behave as rigid bodies and perform allosteric motions that are a special case of allostery (see Motlagh et al. 2014 for review).

The comparison of communities and critical residues indicates that allosteric pathways of MBS homomers are characterized by a much higher percentage of residues in MCCs and critical residues in interfaces than SBS homomers, due to their higher relative interface area (figs. 2 and 3). This indicates that in MBS homomers, allosteric communication typically involves pathways that cross protein–protein interfaces, whereas in SBS homomers, this is much less frequent and the subunits of the complex behave largely independently. Additionally, ligand binding in MBS homomers results in a different degree of change in the percentage of residues in MCCs compared with SBS homomers (fig. 5), suggesting qualitative differences in the dynamics of conformational changes upon ligand binding. Although in this work we analyzed only the community structure of allosteric complexes, the high frequency of MCCs in most MBS homomers, which enable efficient information transfer between the subunits of the complex, might be one of the key properties that facilitates the evolution of allostery. Taken together, these differences in allosteric pathways suggest that in MBS and SBS homomers, the allosteric changes are qualitatively different. In SBS homomers, the evolution of quaternary structure is less likely to be influenced by the biochemical function of the protein, but more by neutral processes (Lynch 2013; Abrusán and Marsh 2018; Hagner et al. 2018), and other factors like the regulation of their degradation (McShane et al. 2016; Mallik and Kundu 2018). Our analysis of interface conservation does not suggest that in SBS complexes nonobligatory or transient interactions are more frequent (supplementary fig. S5, Supplementary Material online), whereas their different community structures, smaller interfaces, and less conserved quaternary structures (Abrusán and Marsh 2018) do support this. Since the currently available experimental data on the obligatory or nonobligatory nature of homomers is much less abundant than for heteromers, further studies are necessary to clarify this. Finally, the comparison of the residue c.o.m. and C-α atom-based methods for the identification of communities and critical residues indicates that the former performs somewhat better in the identification of biologically relevant residues, supporting the conclusions of VanWart et al. (2012) (fig. 7).

The modern view of allostery is based on the dynamic ensemble properties of proteins (Kornev and Taylor 2015; Dokholyan 2016). However, the classic MWC and KNF models continue to be used, particularly for symmetric homomers, due to their simplicity (Cornish-Bowden 2013). Our results suggest that in the case of SBS homomers, the KNF model probably provides a better approximation of reality, because, no particular symmetry type appears to be highly enriched among such allosteric complexes (fig. 1 and supplementary fig. S1, Supplementary Material online), although the high frequency of cyclic symmetry in all homomers makes it very likely that the MWC model also holds for many cases. Additionally, in the case of SBS homomers, ligand binding results in a reduction of residues in MCCs (fig. 5*A*), suggesting that allosteric motions in these proteins are primarily the result of structural changes in individual subunits, and the classic hemoglobin like “tense”—“relaxed” transitions apply primarily to these complexes (best seen with the residue c.o.m. method, fig. 5*A*). The case of MBS homomers is probably more complex. The enrichment of dihedral symmetry (fig. 2*G*, see also Goodsell and Olson 2000; Bergendahl and Marsh 2017) suggests that, for some of these complexes, the MWC model might be a good approximation. However, the high frequency of MCCs in such complexes (i.e., the fact that their “quasi-rigid” communities involve residues from multiple chains), both in their apo and holo structures suggest that, for most of them, neither the MWC nor the KNF model is adequate, and such complexes behave largely as a single monomeric unit.

Research on allostery has traditionally focused on the mechanics and structural characteristics of allosteric changes. Surprisingly, the equally fundamental question “What are the driving forces behind the evolution of allostery?” has received much less attention. It has been suggested (Gunasekaran et al. 2004) that allostery might be a property of every dynamic protein. In our opinion, this is probably an overly broad view of allostery. Although it cannot be excluded that for most dynamic proteins, it is possible to design an allosteric modulator of some sort, there is likely to be a substantial difference between such “ad hoc” allostery and proteins whose allosteric motions have evolved for millions of years, and are actively used by the cellular machinery. The evolution of allostery has been studied more in proteins where the “domino model” is the predominant mode of allosteric signal transduction. It has been suggested that allosteric pathways preexist in such proteins within so-called “sectors” (Reynolds et al. 2011), and that fundamentally allostery is related to the evolvability of proteins—thus it is a consequence of residue coevolution, and is not the consequence of the necessity to be regulated (Raman et al. 2016; Pincus et al. 2017). The generality of these hypotheses remains to be seen (and whether they are applicable to proteins where the “violin model” of allostery is the predominant one), however, our analysis of binding-site similarity supports the idea that the need to be regulated is a significant driving force in the evolution of allostery (fig. 6 and supplementary fig. S9, Supplementary Material online). Our results indicate that having less specific binding sites (potentially resulting in off-target activity) is an important factor facilitating the emergence of allosteric regulation (fig. 6). Since allosteric proteins of all quaternary structure types (except MBS homomers) have either more homologs with similar binding site, or a higher likelihood of having a homolog with a similar binding site, it appears to be a general force shaping the evolution of allostery. However, the current limitations of the PDB mean that there is considerable uncertainty about the magnitude of the effect. Although the human proteome has the best coverage in the PDB, only 33.8% of human proteins have a structural entry (including homologs with >90% sequence identity), and since structures frequently cover only fragments or domains of proteins, the actual sequence coverage of the human proteome is only 16.8%. (These numbers are considerably higher when structures with reliable homology models are included, though [Xie et al. 2011].)

## Materials and Methods

### Determination of Protein Complexes, Ligand-Binding-Site Structure, and Ligand Type

Protein complexes were determined as follows. First, we downloaded the uniprot-pdb mappings (cross-ref) from the UniProt database. Allosteric proteins were defined as proteins present in the Allosteric Database (ASD) v3 (Shen et al. 2016: 3). Using the first biological assembly of each PDB entry, we determined the quaternary structure of the proteins. Proteins in the PDB frequently have multiple entries, and the quaternary structure of these entries may differ, i.e., the same protein can have a homomeric, monomeric, or part of heteromeric structures. We used the following: hierarchical protocol to determine the quaternary structure for every protein in the Allosteric Database: if the protein is part of at least one heteromeric complex, it was classified as heteromer; else, if it has at least one homomeric structure, it was classified as a homomer; the remaining proteins were classified as monomers. Proteins that have a hetromeric entry were not included in the homomer or monomer data sets, even if they have homomeric or monomeric entries. In the case of heteromers, their entries contain several different proteins, and the same protein may be part of different complexes. To account for redundancies, if two or more proteins were part of the same heteromeric entry, the structure was used only once.

Ligand-binding-site structure was determined as described previously (Abrusán and Marsh 2018), using the BioLiP database (Yang et al. 2012). We focused on small-molecule ligands, and excluded nucleic acid or peptide ligands from the analysis. Complexes having binding sites with residues originating from several different chains were classified as MBS complexes, and all others complexes were classified as SBS complexes. We excluded several PDB entries from BioLiP that cannot be seen as a “molecular machine,” that is, that are part of a virus (mostly capsids), form protein fibrils, are helical, contain ubiquitin, or chains without an interface. We also excluded all entries where the biological assemblies contain chains absent in the asymmetric unit, or the difference between the asymmetric unit and biological assembly affects the classification of the binding site. This was necessary because BioLiP is based on the asymmetric units.

The type of each ligand in the PDB structures (allosteric vs. orthosteric, allosteric inhibitor vs. activator) was determined using the classification of ASD. First, we determined the InChiKey of every allosteric modulator of a given protein in the ASD with OpenBabel (O’Boyle et al. 2011), using their 3D (mol2) structure provided by ASD. Second, we downloaded the SDF files of ligands from the PDB, extracted their InChiKeys, and tested whether they are present among the allosteric modulators of a given protein in ASD. Ligands that were not present among the allosteric modulators were classified as orthosteric ligands, whereas the ones that are present in ASD were assigned their ASD classification (i.e., activator, inhibitor, or regulator).

The symmetry group of every PDB entry was taken directly from the symmetry assignments of the first biological assemblies present in the PDB. In the case of heteromers, “monomeric” symmetry represents complexes with 1: 1 stoichiometry, that is, where every protein has only a single copy in the complex. Asymmetric heteromers were those with at least one repeated subunit that are also classified as asymmetric by the PDB. Since a single complex or protein typically has several entries in the PDB, the symmetry of complexes was defined with the following protocol. First we determined the symmetry of every PDB entry that is part of a given complex (in the case of homomers this means every PDB entry that maps to a given UniProt sequence). Next, we applied the following hierarchy in determining the symmetry type: Dihedral -> Higher-order cyclic, i.e. cyclic with, for example, 3 or more subunits (CyclicN)-> Two-fold symmetric (Cyclic2) -> Monomeric -> Asymmetric. Thus, if the structures of a complex have at least one dihedral entry then it was assigned dihedral (irrespectively whether, and how many entries with different symmetry it has); if no dihedral structure exists but there is minimum one CyclicN structure, then we assigned it as CyclicN, and so on.

### Selection of Ligand-Binding Structures for Allosteric Pathway Identification

For each allosteric complex identified above, a single representative structure was chosen for the analysis with STRESS. When multiple entries were present for the same complex we selected the entry for analysis with a following decision tree: resolution better than 2.8 Å -> (if there is more than one such entry) entry has the largest number of proteins and chains -> entry has the largest ligand -> entry has the best resolution. In general we selected the largest structures, with the largest possible ligand and with the best resolution. The list of structures used in the analysis is available in supplementary table S1, Supplementary Material online.

Homomeric structures used in the apo-holo comparisons were selected as follows. First, we determined clusters of PDB entries that are associated with the same PubMed ID with their protein sequences present in the Allosteric Database. Next we kept only those clusters that have at least one entry that is absent in the BioLiP database (apo structures), and a minimum of one that is present in the BioLip database (holo structures). For both the apo and holo structures, we applied the same selection procedure as described above to choose the largest valid structure with the highest resolution and largest ligand (for holo structures). Additionally, the apo–holo pairs were required to have identical numbers of chains, and we also excluded pairs where the length of sequence in their actual structures differs more than 10%. Since holo structures are sometimes crystallized with several different ligand types (orthosteric ligands or allosteric modulators), one apo structure is sometimes associated with more than one holo structure (e.g., if structures with an orthosteric ligand and with an allosteric inhibitor are both present). Only a single structure was allowed for each ligand type (if present). The list of apo–holo pairs used in the analysis, and their ligand types are available in supplementary table S2, Supplementary Material online.

### Identification of Communities and Critical Residues with STRESS

Before running STRESS, the structures were preprocessed. First, peptide ligands (as defined by BioLiP [Yang et al. 2012]) were removed from the structures; second, the structures were processed with the dock-prep tool of Chimera (Pettersen et al. 2004) to complete incomplete side chains, add hydrogens, and remove residues with low occupancy when residues with alternative locations are present. Next, we modified STRESS to use residue c.o.m. in the identification of communities, instead of C-α atoms. Using the Bio3D R package (Skjærven et al. 2014) and in house Perl scripts, we calculated the residue c.o.m. for every residue in the structure, and substituted the *_CA.pdb file produced by STRESS, containing the coordinates of C-α atoms with a file with a similar format, but containing the coordinates of the residue c.o.m. of each residue. Finally, we ran STRESS with the “-interior” flag using the preprocessed structure, to identify communities and interior critical residues. We also processed every structure with the unmodified version of STRESS. A community was defined as a MCC if its residues are distributed between minimum two chains of the complex, and more than 10% of its residues fall to each chain. (Thus, cases when 99 of a 100 residue community fall into one chain and a single residue falls into another one were not classified as MCCs.)

### Calculation of the Energetic Contribution of Individual Residues to the Binding Energy of Interfaces

We used FoldX (Schymkowitz et al. 2005) to estimate the independent energetic effects of residues. First, using the structures preprocessed for STRESS, we ran the RepairPDB FoldX module, to correct van der Waals clashes and torsion angles in the structure. Next, we determined the binding energies of each interface pair in the complex with the AnalyzeComplex module. Third, we mutated every residue individually in protein–protein interfaces to alanine (except alanines), and re-calculated the interface binding energies in the mutant structures. The difference between the mutant structures and the original structure gives an estimate of the contribution of the residue to the binding energy between different chains. Since binding energies are negative, a positive effect means weakening of binding between the interfaces due to a mutation.

### Calculation of Relative Solvent Accessibilities

Relative solvent accessibility (RSA) was calculated using the entire protein complex (first biological assembly); thus, residues buried in interfaces can have RSA of zero. For each complex, the solvent accessible surface of every residue was determined using DSSP (Carter et al. 2003). Next the solvent accessibilities were normalized with the solvent accessibility of the amino acid in a three amino acid peptide that mimics the solvent accessibility in an unfolded protein (Miller et al. 1987). Finally, the average RSA was calculated using all interface residues of a community.

### Calculation of Conservation Scores

Conservation scores for each protein were calculated with a pipeline that was conceptually similar to (and modeled on) the conservation score calculations in the ConSurf Database (Goldenberg et al. 2009). First, we identified homologs to the sequences of the PDB entries in a filtered UniRef90 database with CS Blast (Biegert and Söding 2009), using three iterations and an *e*-value cutoff of 0.0001. During filtering, we removed all entries from Uniref90 where the fasta header contains the words “hypothetical,” “undetermined,” “whole genome shotgun sequence,” “fragment,” “mutant,” “mutation,” and “variant.” This reduced the number of entries in UniRef90 from ∼70 million to ∼52 million. Next, the sequences of the significant hits were clustered with UCLUST (Edgar 2010), with 90% identity cut-off. The cluster centroids with the highest *e*-values were then aligned with MUSCLE (Edgar 2004), and the conservation scores of the alignments were calculated with Rate4Site (Pupko et al. 2002), with the empirical Bayesian method. A maximum of 250 sequences was used, and a minimum of 50 sequences was required in the evolutionary rate calculations. Since Rate4Site provides evolutionary rates, we used the inverse of rate as the conservation score; thus higher values indicate higher conservation. In the final step of the pipeline, we mapped the sequence of the actual PDB structure (that can differ at individual positions, or can contain gaps) to the sequence in the PDB_seqres file by making pairwise alignments with MUSCLE.

### Interface and Surface Area Determination

The total solvent accessible surface area formed by each polypeptide chain was calculated using AREAIMOL from the CCP4 suite (Winn et al. 2011). The interface area was calculated as the difference between the solvent accessible surface area of each subunit in isolation and within the context of the full complex. Subunit flexibility was calculated on the basis of relative solvent accessible surface area (*A*_rel_), as described previously (Marsh and Teichmann 2011).

### Identification of Homologs in the Human Genome with Similar Binding Sites

We used a pipeline that was largely similar to the one used in our previous study (Abrusán and Marsh 2018), with some modifications. First, we identified homologs of human proteins in the PDB among the proteins present in PDB with BlastP, with an *e*-value cut-off of 10^−5^, up to 10,000 hits. Next, using the query sequences that have a ligand-binding structure in the BioLiP database, we performed an exhaustive search for similar binding pockets, using the ligand-binding pockets of all structures of the query sequence against all structures of all homologous target sequences, including the structures that have no ligand, using ProBis (Konc and Janežič 2010; 2017). Proteins with chimeric PDB entries were excluded from the analysis. Binding sites were defined as the residues within 3 Å of the ligand; hits with *Z*-score above 2 (calculated by ProBis) were accepted as significant if the hit contains residues from a sequence that is homologous to the query sequence (i.e., the target is homologous to the query; in the case of hits to heteromer structures this is not always the case). Homologous sequence pairs with a similar binding site were defined as sequences that have at least one shared binding site, i.e., that either have a query or a target sequence with a significant hit.

### Calculation of Evolutionary Couplings

First, in every protein sequence of the allosteric structures, we identified the nonoverlapping Pfam (Finn et al. 2016) domains with hmmscan (Eddy 2011), with an *e*-value cut-off 0.001. Next using the sequences of the identified Pfam domains, we identified (and aligned) homologous sequences in the UniRef100 database with jackhammer (Eddy 2011), with an *e*-value cut-off 0.001, and five iterations. The homologous sequences were clustered at 90% sequence similarity and 75% sequence coverage with usearch (Edgar 2010). Additionally, we removed every sequence with less than 75% overlapping residues with the query, and trimmed the alignments to the query sequence, so the final alignments contained only columns from the query domain sequence. If the final alignments contained more sequences than five times the length of the query domain, we ran GREMLIN (Kamisetty et al. 2013) (C++ version, provided by S. Ovchinnikov), to identify evolutionary couplings between the residues. Before calculating the strength of couplings between critical and community residues, the AP corrected Frobenius scores reported by GREMLIN were standardized with the standard deviation of the scores within each domain, to correct for differences between domains. For every structure, the average coupling score was used, both for critical and community residues.

## Visualization and Statistics

All statistical tests were performed with in-house Perl scripts and R. Protein structures were visualized with PyMol (v1.7.6.0, open source version).

## Data Availability

Additional supplementary dataset is available at https://datashare.is.ed.ac.uk/handle/10283/3253.

## Supplementary Material

Supplementary data are available at *Molecular Biology and Evolution* online.

## Supplementary Material

msz093_Supplementary_Data
