# Supplementary material for: Ligand-Binding-Site Structure Shapes Allosteric Signal Transduction and the Evolution of Allostery in Protein Complexes
Source: Mol Biol Evol. 2019 Apr 19;36(8):1711–27. doi: 10.1093/molbev/msz093 (PMC6657754; doi:10.1093/molbev/msz093)
Supplement: msz093_Supplementary_Data [file msz093_supplementary_data.zip › SuppFigures.pdf]

SUPPLEMENTARY FIGURES

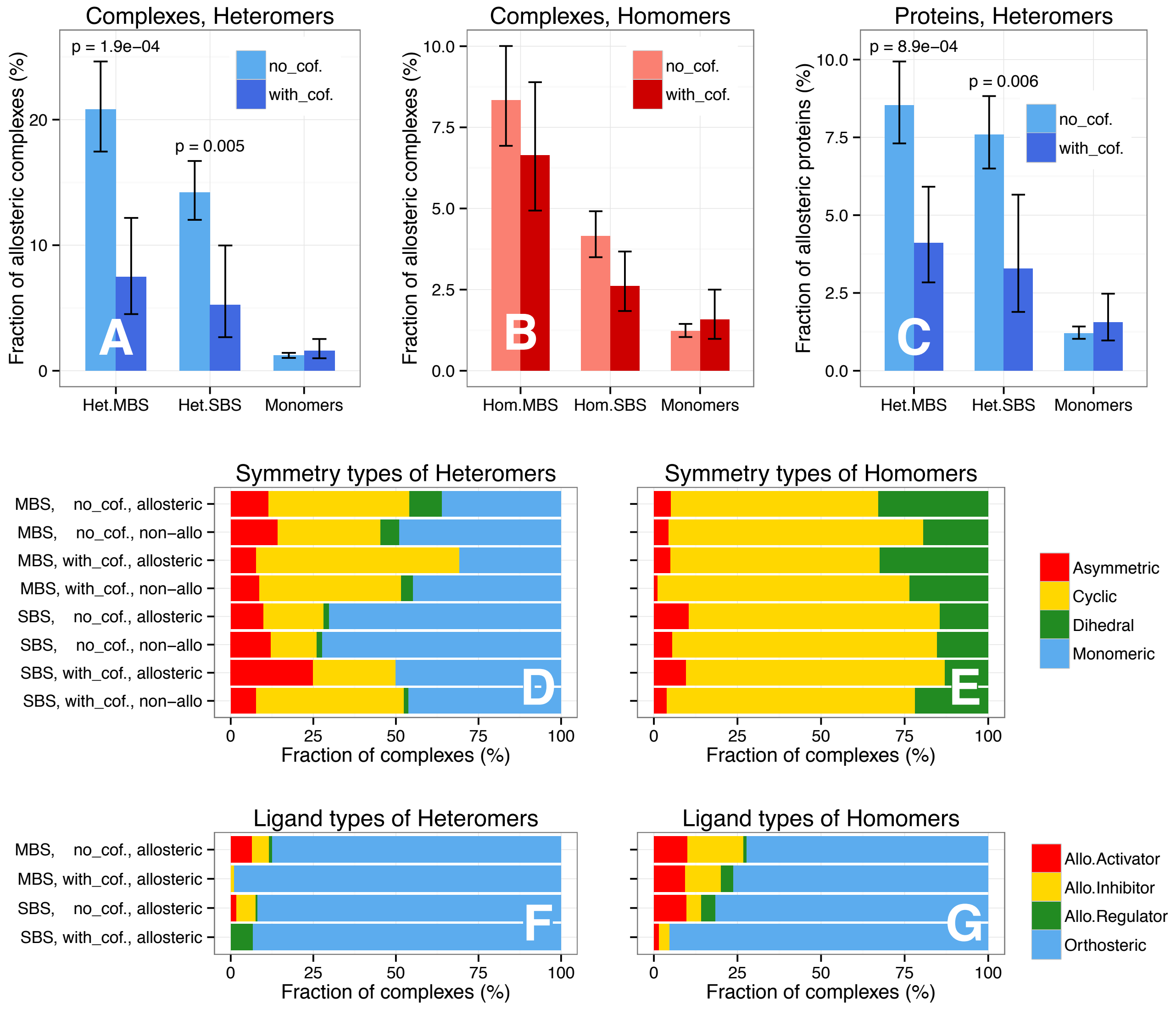

Figure S1. Allostery is less frequent in heteromeric complexes binding cofactors. Both in the case of complexes (**A**), and proteins (**C**), the frequency of allostery is significantly lower among the cofactor binding ones (all tests are tests of proportions). In homomers the differences are not significant, although a similar tendency appears to be present (**B**). Similarly to metal binding, the differences are unlikely to be caused by differences in symmetry (**D and E**), or ligand types (**F and G**), although the higher frequency of monomeric symmetry in SBS heteromers without cofactors may contribute to the difference (**D**).

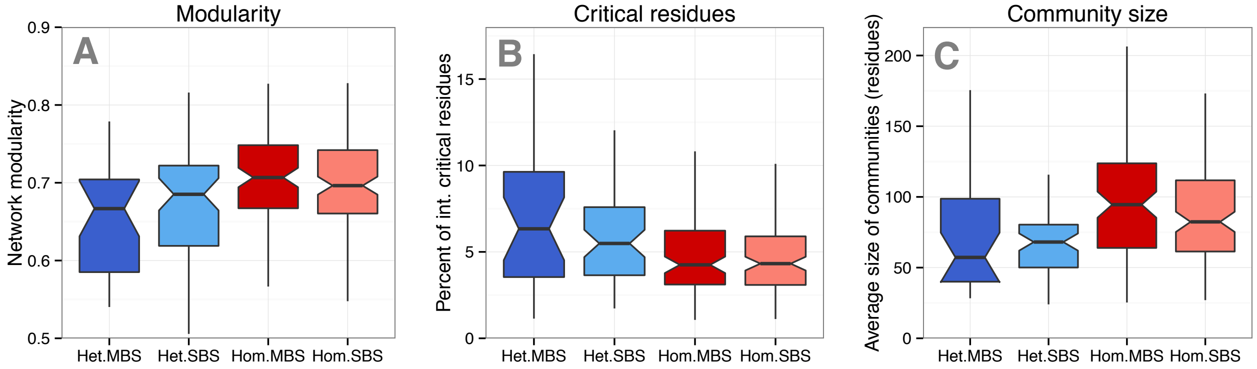

Figure S2. Characteristics of the residue networks and communities in allosteric protein complexes. **A**) Network modularity, **B**) Percent of interior critical residues, **C**) Average size of communities. Homomers and heteromers are characterized by somewhat different values, but there are no dramatic differences between MBS and SBS structures.

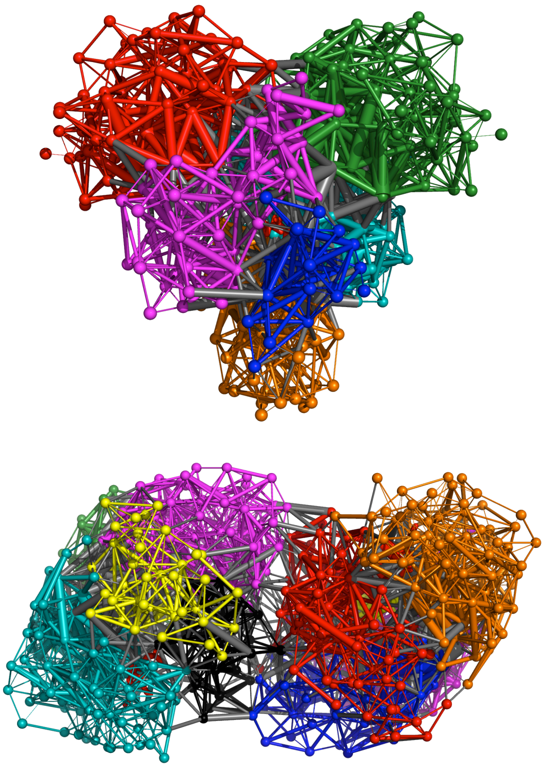

Figure S3. Community structures of the Global Nitrogen Regulator Protein (MBS homomer, PDB ID: 3la3, top), and UDP-Galactose 4-epimerase (SBS homomer, PDB ID: 5gy7, bottom).

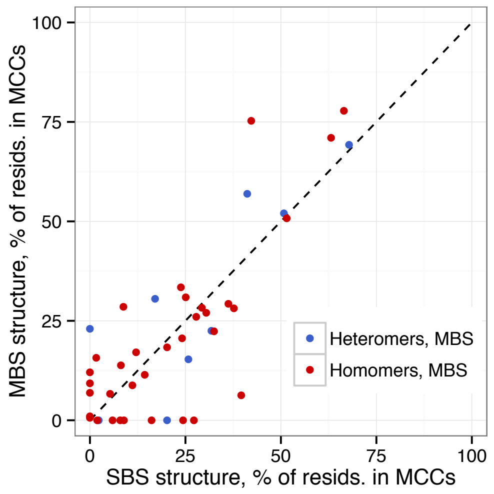

Figure S4. Protein complexes that have both an MBS and SBS structure in the PDB have largely similar fraction of residues in MCCs (the dashed line is x = y). This raises the possibility that for SBS complexes the fraction of residues in MCCs is overestimated, because for certain complexes their MBS form is probably missing from the PDB.

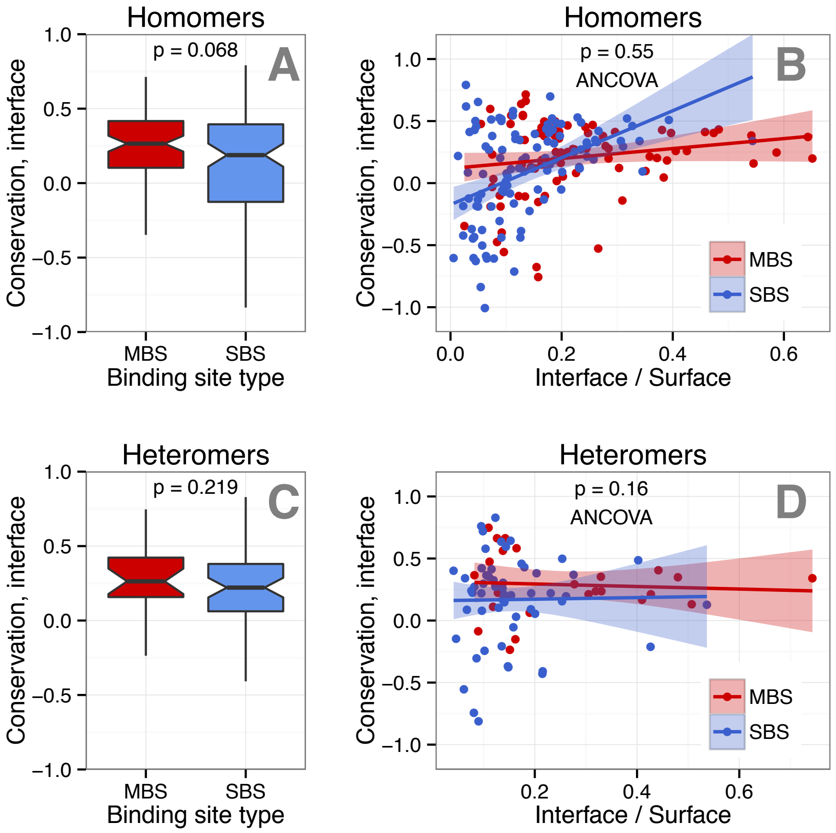

Figure S5. Interface conservation in homomers and heteromers. We found no significant differences between the conservation of interface residues of MBS and SBS complexes, and even the nonsigificant trend in homomers (panel A) disappears when relative interface area is used as covariate (panel B).

Wild type, ASP, B:162 (cyan)

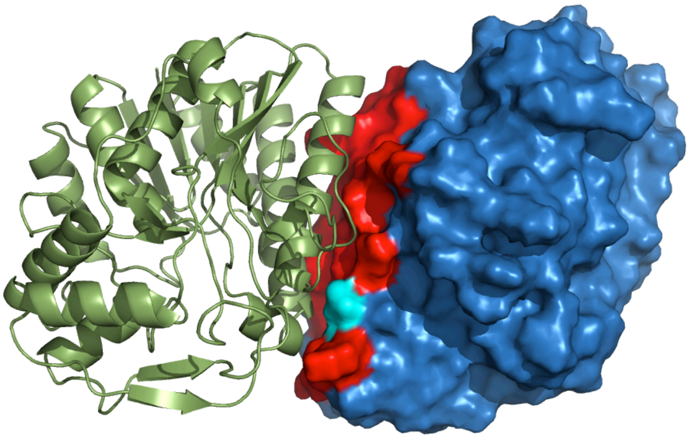

Interface binding energy: -30.4038 kcal/mol

Mutant, ALA, B:162 (yellow)

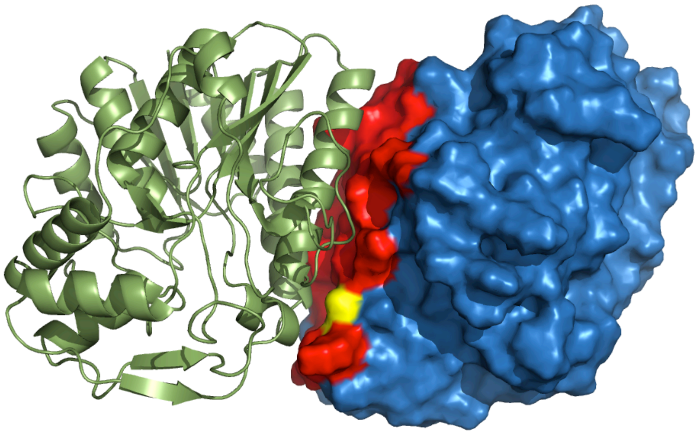

Interface binding energy: -28.1846 kcal/mol
Difference = 2.2192 kcal/mol

Figure S6. Calculation of the effect of interface mutations on binding energies. Upper panel: wild type structure of UDP-Galactose 4-epimerase dimer (PDB ID: 5gy7). On chain B the surface is indicated with blue, and the interface with red. The interface residue ASP B:162 is highlighted with cyan. The energy of the interface formation is -30.4038 kcal/mol. Lower panel: the ASP162ALA mutant of UDP-Galactose 4-epimerase, with ALA B:162 highlighted with yellow. The energy of the interface formation is -28.1846 kcal/mol, thus the mutation weakened the interface binding energy with 2.2192 kcal/mol.

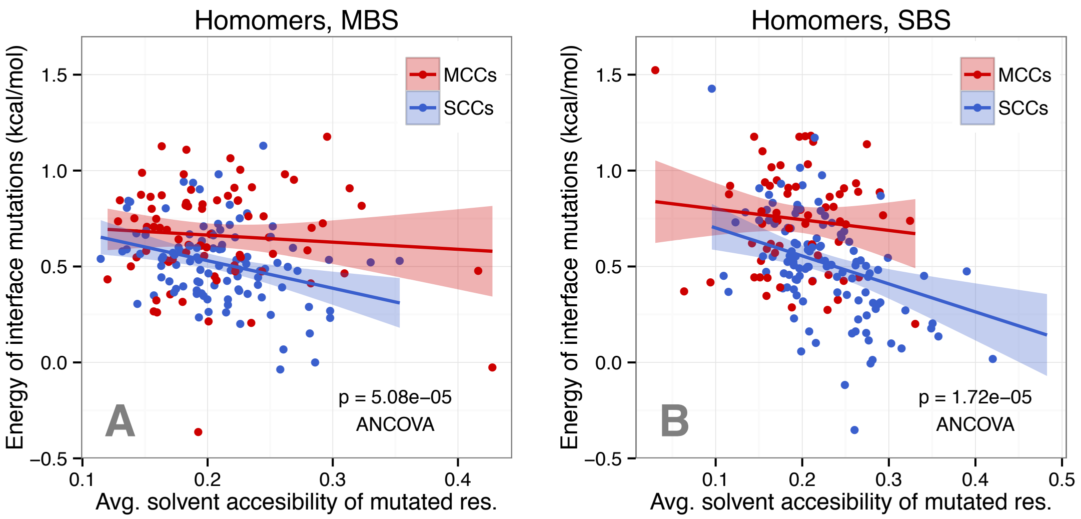

Figure S7. The difference between MCCs and SCCs remains highly significant when solvent accessibility is added as a covariate, indicating that the difference between the two types of communities is not a simple by-product of their different location in the interfaces. Note that in the case of SBS homomers 3 outliers with energies below -1,-3 and -3 were excluded.

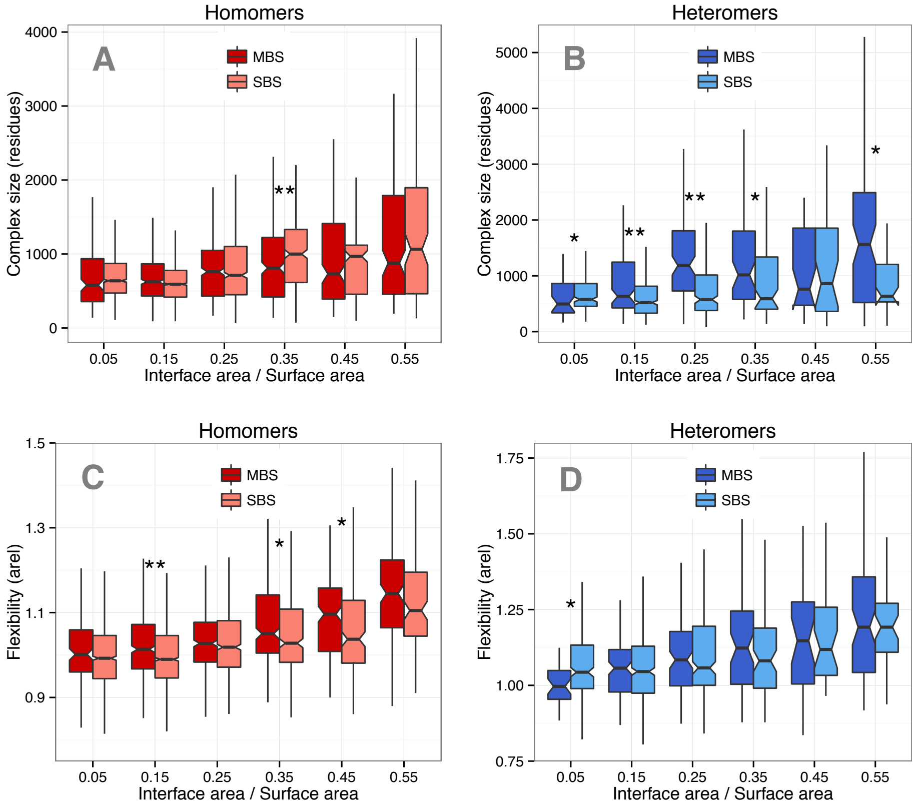

Figure S8. The size of protein complexes increases with increasing interface to surface ratio, both in homomers (**A**) and heteromers (**B**). The flexibility of protein chains also increases with increasing interface to surface ratio (**C** and **D**), but the differences within each bin do not seem to be sufficient to explain the differences in the frequency of allostery. (All tests are Wilcoxon tests.)

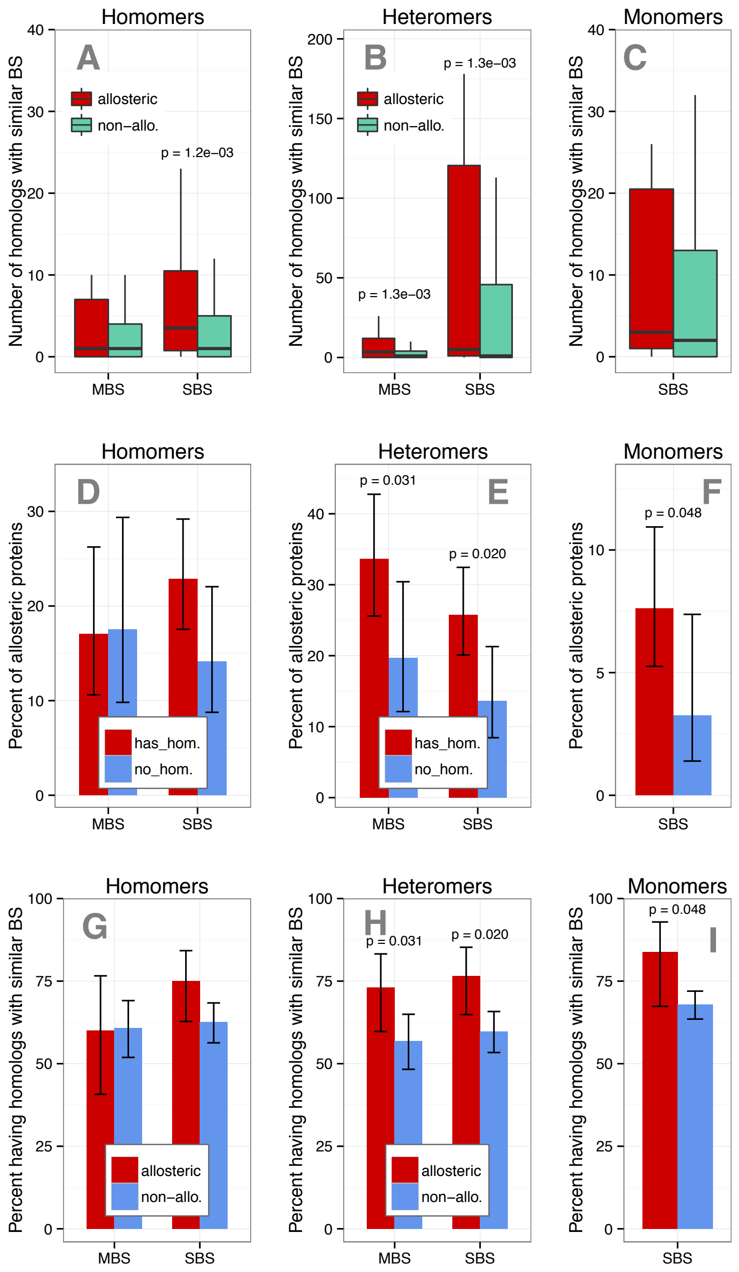

Figure S9. The relationships between the number/presence of homologs with similar binding site (BS), and allostery do not change qualitatively when metal and cofactor ligands are excluded from the analysis (and in consequence proteins having only metal and cofactor ligands). While significances are generally weaker, partly due to the smaller number of proteins (one sided Wilcoxon tests**,** panels **A-C,** one sided tests of proportions, panels **D-I**), the overall pattern and effect sizes are similar to Figure 6; i.e. proteins with (more) homologs with similar BS are more likely to be allosteric.
